# A novel role of PRR14 in the regulation of skeletal myogenesis

**DOI:** 10.1038/cddis.2015.103

**Published:** 2015-04-23

**Authors:** M Yang, Z-M Yuan

**Affiliations:** 1 Department of Genetics and Complex Diseases, Harvard T.H. Chan School of Public Health, Boston, MA, USA

## Abstract

Dysregulation of genes involved in organizing and maintaining nuclear structures, such as SYNE1, SYNE2, TREM43, EMD and LMNA is frequently associated with diverse diseases termed laminopathies, which often affect the muscle tissue. The PRR14 protein was recently reported to tether heterochromatin to nuclear lamina but its function remains largely unknown. Here, we present several lines of evidence demonstrating a critical role of PRR14 in regulation of myoblast differentiation. We found that Prr14 expression was upregulated during skeletal myogenesis. Knockdown of *Prr14* impeded, whereas overexpression of PRR14 enhanced C2C12 differentiation. The pro-myogenesis activity of PRR14 seemed to correlate with its ability to support cell survival and to maintain the stability and structure of lamin A/C. In addition, PRR14 stimulated the activity of MyoD via binding to heterochromatin protein 1 alpha (HP1*α*). The results altogether support a model in which PRR14 promotes skeletal myogenesis via supporting nuclear lamina structure and enhancing the activity of MyoD.

The nuclear lamina is a scaffold-like network of protein filaments surrounding the nuclear periphery.^1, 2, 3, 4, 5^ As an essential component of metazoan cells, it is involved in most nuclear activities, including DNA replication, RNA transcription, nuclear and chromatin organization, cell cycle regulation, cell development and differentiation, nuclear migration, and apoptosis.^6, 7^ The nuclear lamina is composed of lamins, which are also present in the nuclear interior, and lamin-associated proteins. Specific mutations in nuclear lamina genes cause laminopathies, a group of rare genetic disorders presenting a large variety of clinical symptoms in different tissues. These diseases include Emery–Dreifuss muscular dystrophy,^4, 8^ Atypical Werner syndrome,^9^ dilated cardiomyopathy,^10^ Buschke–Ollendorff syndrome,^11^ Limb-girdle muscular dystrophy,^12^ Hutchinson–Gilford progeria syndrome,^13^ Barraquer–Simons syndrome,^14^ Charcot–Marie–Tooth disease,^15^ Pelger–Huet anomaly,^16^ Pelizaeus–Merzbacher disease,^17^ Tight skin contracture syndrome^18^ and so on. Among all the different clinical presentations, muscle seems to be the most common target of laminopathies.

The lamin family of proteins consists of the matrix of nuclear lamina. There are three types, A, B and C, of lamins in humans, encoded by three genes: *LMNA* for A-type lamins (lamin A/C are encoded by the *LMNA* gene through alternate splicing), *LMNB1* for lamin B1 and *LMNB2* for lamin B2. In contrast to B-type lamins, which are ubiquitously present in all cell types, A-type lamin expression is largely restricted to differentiated tissues, particularly skeletal muscle. Among all the nuclear lamina components, *LMNA* is the first and most studied gene associated with laminopathies. Various mutations in *LMNA* have been reported to result in muscular dystrophies.^4, 12, 19, 20, 21^ The mutations are located throughout the gene and they impair myogenesis either by resulting in mechanical weakness of the nuclear lamina and of the cells, especially in striated muscle cells,^22^ or by regulation of gene expression through direct interactions with different transcription factors and chromatin-modifying complexes.^23^ One of the critical functions of the nuclear lamina is to organize heterochromatin,^24^ which, as part of nuclear chromatin architecture, has an important role in nuclear organization. Along with the nucleus, heterochromatin undergoes a global reorganization during myogenesis. Heterochromatin protein 1 isoform, a constitutive component of heterochromatin, is also implicated in myogenesis.^25, 26^

Proline rich 14 (PRR14), a not well-studied protein, was recently reported to function as a structural protein that tethers heterochromatin to nuclear lamina through functional associations with HP1*α* and the nuclear lamin A/C.^5^ Here, we provide evidence that uncovers a novel function of PRR14 in regulation of myoblasts differentiation and interestingly, PRR14 directly binds to HP1*α* and this binding has an important role during myogenesis.

## Results

### RNAi-mediated depletion of Prr14 inhibits myoblast differentiation

**Figure 1:**
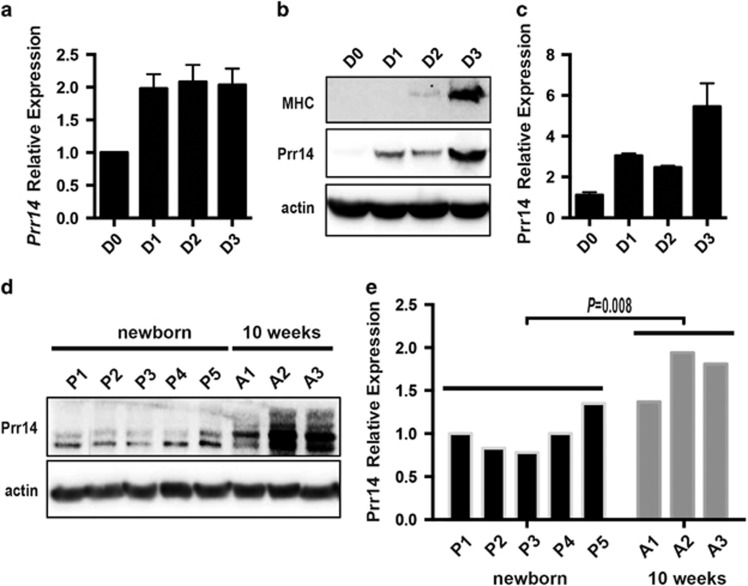
Prr14 is upregulated during skeletal muscle differentiation. C2C12 cells were plated in GM. After 24 h, differentiation was stimulated by changing the medium into DM. Cells were harvested at indicated time points and the mRNA and protein level was detected by qRT-PCR (**a**) and immunoblot (**b**), respectively. (**c**) The level of PRR14 protein was quantified. The numbers of relative abundance of both mRNA and protein are showed and presented as the mean±S.D. (*n*=3). (**d**) Immunoblot analysis of Prr14 in gastrocnemius skeletal muscle obtained from newborn (*n*=5), as well as 10-week-old (*n*=3) mice. (**e**) The protein levels were quantified and two-tailed student's *t-*test was used to determine the significance of the difference between the two groups

PRR14 was reported to be functionally related with lamin A/C,^5^ which is implicated in myogenesis.^4, 12, 19, 20, 21^ We asked whether PRR14 might contribute to the regulation of myogenesis. To address this question, we measured the expression of *Prr14* during the course of C2C12 myoblast differentiation. Using quantitative RT-PCR analysis, we observed that the *Prr14* transcript level was indeed increased at day 1 upon differentiation induction and sustained thereafter (Figure 1a). The upregulation of Prr14 was confirmed at the protein level, which co-related with the induction of myosin heavy chain (MHC), a surrogate marker of the skeletal muscle differentiation (Figures 1b and c). It has been demonstrated that the differentiation of skeletal muscle continues after birth until the adult stage.^27^ We collected mouse gastrocnemius muscle from newborn and 10-week-old mice (C57BL/6 J, 000664, JAX) to determine the expression of PRR14. The result showed that the PRR14 protein level in the skeletal muscle is significantly higher in 10-week-old mice than in newborn mice (Figures 1d and e). Collectively, both *in vitro* and *in vivo* data revealed an increased PRR14 expression during skeletal muscle differentiation.

**Figure 2:**
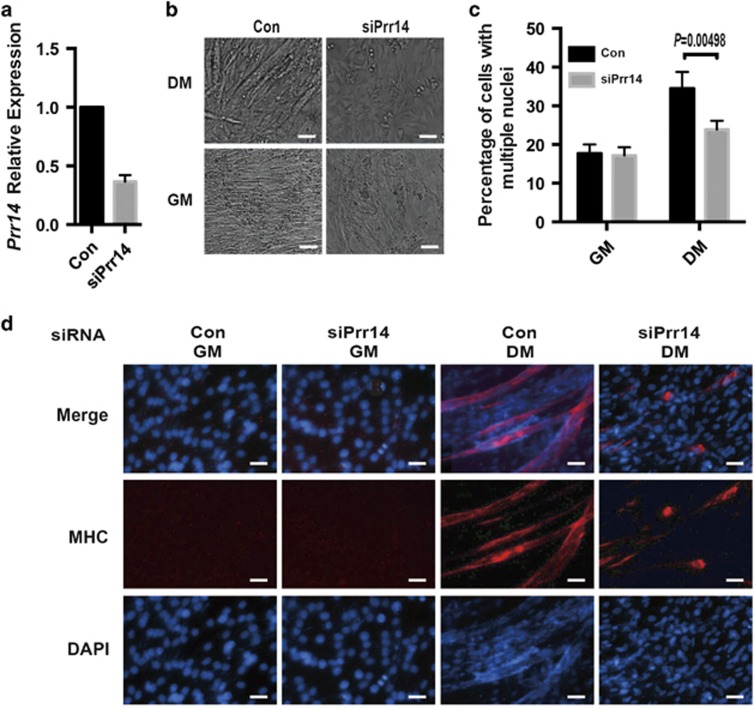
Prr14 underexpression impeded myotube formation in C2C12 cells. C2C12 cells were transfected with the indicated siRNA in GM. At 24 h post-transfection, cell media was replaced with DM and cultured for 5 days. (**a**) Transcripts of *Prr14* were detected by qRT-PCR with 18 s as an internal control. The numbers were presented as mean±S.D. (*n*=3). (**b**) Phase-contrast images of cells cultured with either GM or DM for 5 days were taken and representative ones are shown. (**c**) FACS analysis of C2C12 cells as in **b** was performed using propidium iodide with FACS Canto II and quantitative determination of percentage of cells with multiple nuclei (>4 N) was shown. The data were presented as mean±S.D. (*n*=3) and statistically analyzed by two-tailed student's *t*-test. (**d**) Immunofluorescence analysis of MHC (red) and DAPI (blue) of the indicated C2C12 cells cultured in either GM or DM for 5-day were carried out. Representative images are shown

To investigate the functional significance of PRR14 induction during myoblast differentiation, we employed the siRNA-mediated method to deplete its expression. A mix of three different siRNAs (SASI_Mm01_00110458, SASI_Mm01_00110459 and SASI_Mm01_00110460, Sigma, St. Louis, MO, USA) targeting three different sites of Prr14 was used to generate Prr14-deficient C2C12 cells. qRT-PCR analysis showed that *Prr14*-specific siRNAs efficiently reduced its mRNA level to about 40%, relative to the non-specific control (Figure 2a; more complete depletion was associated significant cell death is not shown). The cells were assessed for their myogenic potential by culturing in differentiation media (DM) for 5 days. Examination of phase-contrast images revealed that C2C12 cells expressing control siRNA developed into myotubes as expected; however, myotube formation was considerably impaired in cells expressing *Prr14* siRNA (Figure 2b). As C2C12 differentiation is associated with the formation of multinuclear myotube, we measured the cells with multinuclei to quantify the effect of PRR14. By defining the fusion index as the percentage of cells with three or more nuclei, we quantified cells with multinuclei via FACS analysis. The result revealed a significant decrease in the number of multinuclear cells in *Prr14* RNAi-expressing cells relative to the control RNAi cells (24 *versus* 34%, *P*<0.05, Figure 2c). Immunofluorescence analysis of MHC was performed to verify the morphological data. Consistent with the formation of multinuclei myotubes, MHC protein was highly expressed upon induction of differentiation in control RNAi-expressing cells. In contrast, the expression of MHC was markedly diminished in Prr14 siRNA-expressing cells (Figure 2d). The results together indicate that reduced PRR14 expression impeded C2C12-cell differentiation, suggesting a critical role of PRR14 in myoblasts differentiation.

### PRR14 overexpression promoted the differentiation of C2C12 myoblasts

The protein sequence alignment data (available from: http://www.uniprot.org) showed that PRR14 protein sequences are highly conserved during the evolution and the protein sequences between human and mouse share over 72% similarity and 77% positive (Supplementary Figure S1A). When expressed in mouse C2C12 myoblasts, the PRR14 protein is primarily localized in the nucleus and surrounding of the nuclear membrane, with little distribution in the cytoplasm (Supplementary Figure S1B). Of note is the colocalization of PRR14 with the highly dense DAPI-rich structure, suggesting an association of PRR14 in the heterochromatin. This expression pattern is consistent with previously reported data in human cells.^5^

**Figure 3:**
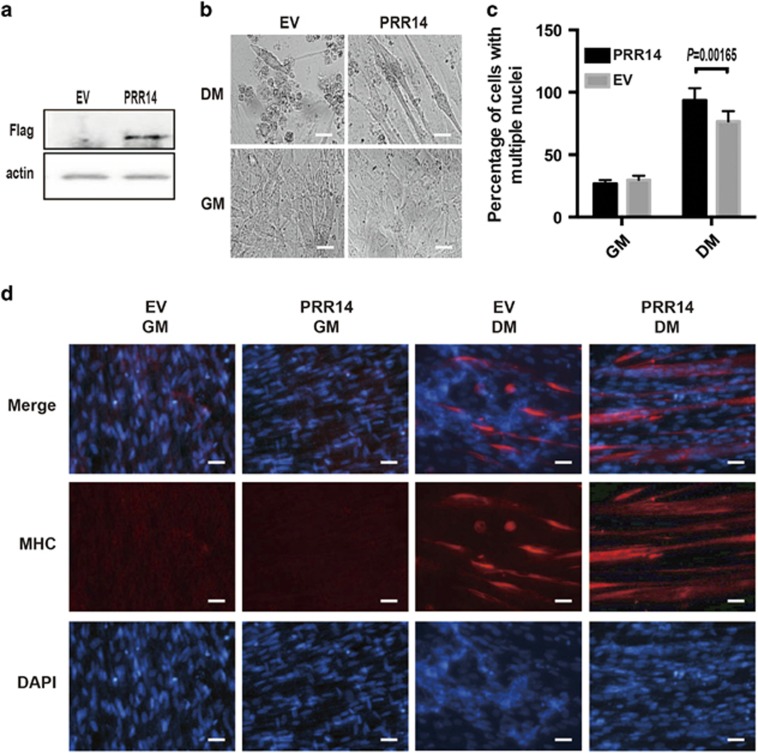
PRR14 overexpression enhances C2C12 myoblasts differentiation. (**a**) PRR14 overexpression in stable cell line was confirmed by immunoblot. C2C12-cell lines were plated and cultured in GM for 24 h, and then cell media was replaced with DM and cultured for 4 days. The representative phase-contrast images are shown (**b**). Percentage of cells with multiple nuclei (>4 N) was quantified by FACS, presented as mean±S.D. (*n*=3) and statistically analyzed by two-tailed student's *t*-test (**c**). The expression of MHC in the indicated C2C12-cell culture in either GM or DM was detected by immunostaining (**d**). The representative images are shown

With the finding that PRR14 underexpression was associated with impediment of C2C12 myoblasts differentiation, we hypothesized that PRR14 overexpression would enhance differentiation. We tested this hypothesis by introducing highly conserved human PRR14 into C2C12 cells. The C2C12-cell line stably expressing PRR14 was established through a retrovirus-mediated gene transfer. The expression of PRR14 was confirmed by immunoblot (Figure 3a). Upon induction of differentiation for 4 days, morphological examination detected more myotubes in PRR14-overexpressing C2C12 myoblasts than the empty vector-expressing control cells (Figure 3b). FACS analysis of the fusion index showed that PRR14-overexpressing C2C12 cells had 93% cells with multinuclei, whereas the control cells had only 76% cells with multinuclei (*P*<0.05, Figure 3c). The enhanced differentiation by PRR14 was further confirmed via MHC immunostaining (Figure 3d). Together, our data indicate that PRR14 overexpression promotes myogenesis.

**Figure 4:**
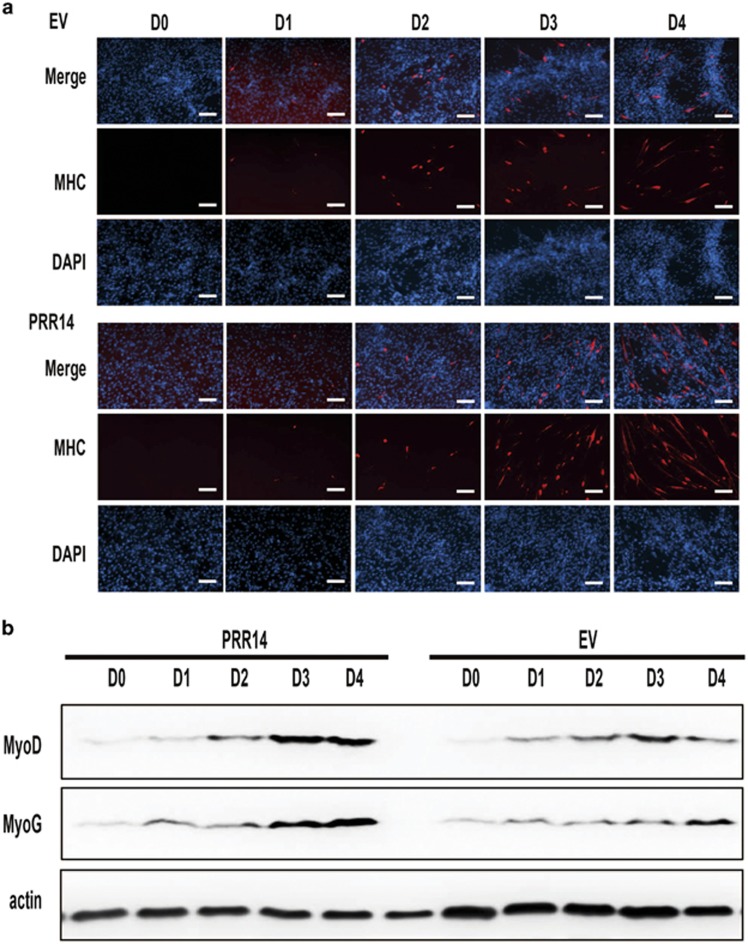
The effect of PRR14 on C2C12 differentiation kinetics. PRR14- or control vector-expressing cell lines were plated and cultured in GM for 24 h, then cell medium was replaced by DM. Cells were collected for either immunostaining (**a**) or immunoblot (**b**) at the indicated time points

To better characterize the role of PRR14 in myogenesis, we performed a time-course experiment to monitor the kinetics of myoblasts differentiation. After culturing in growth medium for 12 h, C2C12 cells were transferred to DM and collected at the indicated time points. Immunostaining of MHC detected its expression at 48 h after incubation in DM. As expected, MHC expression increased as a function of time. Notably the induction of MHC in PRR14-overexpressing cells was markedly increased when compared with the vector-expressing control cells (Figure 4a), consistent with a role for PRR14 in promoting myogenesis. This effect of PRR14 was validated via immunoblot analysis of MyoG, another surrogate marker of myotubes. In agreement with MHC expression, the MyoG protein abundances were higher in PRR14-expressing cells than the control cells (Figure 4b). As both MHC^28, 29^ and MyoG^30^ are direct targets of MyoD during myogenesis in skeletal muscle, which is a major transcription factor regulating the expression of many myogenesis-associated genes, we measured the expression of this transcription factor. Indeed, a higher MyoG expression was correlated with a higher level of MyoD in PRR14-expressing cells suggesting that PRR14 promotes myogenesis via, at least, in part induction of MyoD.

### PRR14 protects cells from apoptosis during myogenesis

**Figure 5:**
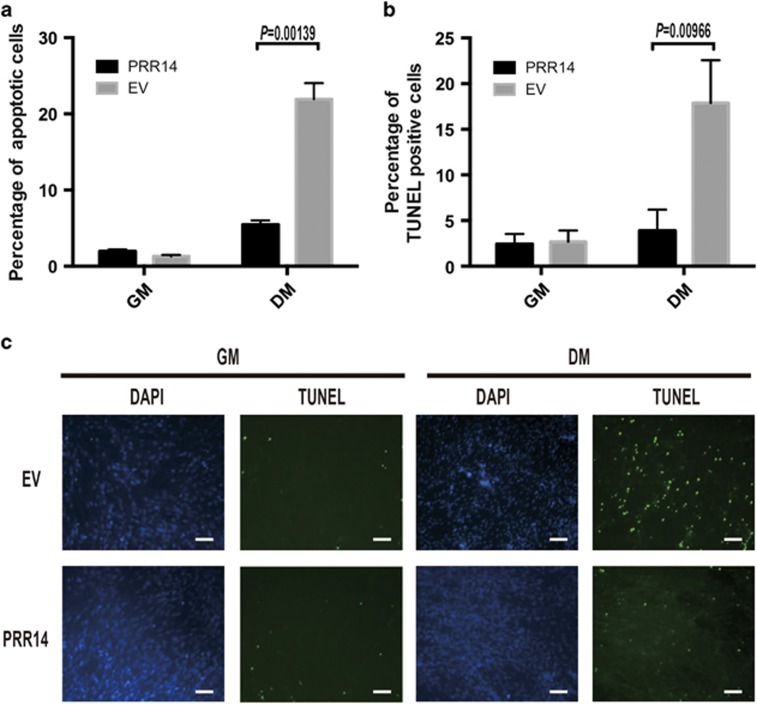
PRR14 protects C2C12 cells from apoptosis during myogenesis. (**a**) Quantitative analysis of sub-G1 population of the differentiated PRR14-expressing and control C2C12 cells via FACS. The data is presented as mean±S.D. (*n*=3) and statistically analyzed by two-tailed student's *t-*test. C2C12 cells stably expressing vector control or PRR14 were subjected to TUNEL assay and counterstained with DAPI (**c**). TUNEL-positive cells were counted in three different fields and 200 cells were counted for each field for quantification. The data are presented as mean±S.D. (*n*=3) and statistically analyzed by two-tailed student's *t-*test (**b**)

While observing myoblast differentiation, we noticed more survival cells in PRR14-expressing cells than the vector-control expressing cells (Figure 3b). To quantify the difference, we used FACS analysis to measure the sub-G1 population. There were 22% apoptotic cells in the control cells and only 6% in PRR14-overexpressing cells during differentiation (*P*<0.05, Figure 5a). To confirm the data, we carried out TUNEL assay (*In Situ* Cell Death Detection Kit, Fluorescein, Roche, Mannheim, Germany). Consistently, there were much less TUNEL-positive cells in PRR14-expressing C2C12 cells (Figure 5b). The result implicates that PRR14 facilitated myoblast differentiation by inhibition of apoptotic cell death.

### Knockdown of *Prr14* was associated with downregulation of lamin A/C and HP1*α*

**Figure 6:**
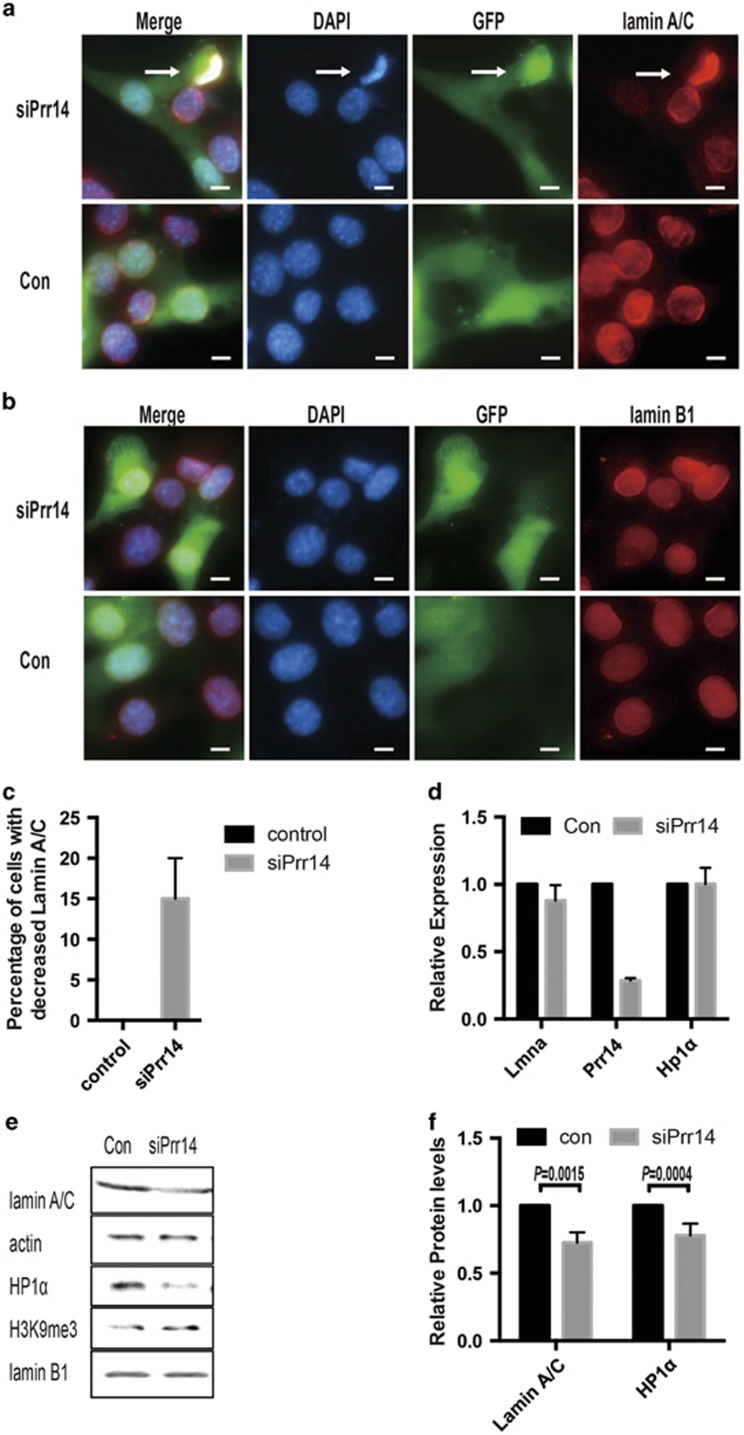
siRNA-mediated *Prr14* depletion is associated with dysregulation of lamin A/C and Hp1*α*. C2C12 cells were co-transfected with siRNA (10 pmol) and EGFP-N1 empty vector (100 ng). Cells were collected 48 h post-transfection and subjected to immunostaining of lamin A/C (**a**) or lamin B1 (**b**) and DAPI. In non-specific control siRNA-transfected cells, lamin A/C was located in either nuclear lamina and cell nucleus, whereas PRR14-siRNA-expressing cells displayed reduced lamin A/C protein level and abnormal nuclear morphology (arrow). (**c**) The percentage of cells with decreased lamin A/C abundance was determined. The numbers are average of three different fields, and 200 cells were counted for each field. The data are presented as mean±S.D. (*n*=3). (**d**) siPrr14 or control siRNA-expressing C2C12 cells were analyzed for mRNA of the indicated genes. The data are presented as mean±S.D. (*n*=3). (**e**) The cells, same as in **d**, were analyzed with immunoblot using the indicated antibodies, and quantifications of WB data are presented as the mean± S.D. (*n*=3) (**f**)

As PRR14 is reported to function as a bridge between lamin A/C and HP1*α,*^5^ we asked whether *Prr14* depletion might have any effect on them, which have been shown to be important during myogenesis. To this end, we examined lamin A/C in *Prr14*-depleted cells. To circumvent the lack of an anti-PRR14 antibody suitable for immunostaining, we co-expressed GFP with siRNA to facilitate the identification of Prr14-deficient cells. Interestingly, siRNA-mediated *Prr14* knockdown in C2C12 cells led to decrease in lamin A/C protein abundance (Figure 6a). Approximately 15% of the cells showed weaker immunostaining (Figure 6c). In addition, *Prr14*-deficient cells also displayed an abnormal nuclear morphology (Figure 6a, arrow). The effect of PRR14 on lamin A/C appeared to be specific as lamin B1, another type of lamin, was not significantly affected under the same condition (Figures 6b and e). Immunoblot confirmed the decrease in lamin A/C, and interestingly the level of HP1*α* was also reduced (Figures 6e and f). qRT-PCR analysis did not detect a significant change in the transcripts of both *lmna* and *Hp1α* (Figure 6d), suggesting that the decrease of lamin A/C and HP1*α* in *Prr14*-deficient cells was caused by a mechanism of post-transcriptional regulation.

### Mutation disrupting the interaction between PRR14 and HP1*α* impairs C2C12 myogenesis

**Figure 7:**
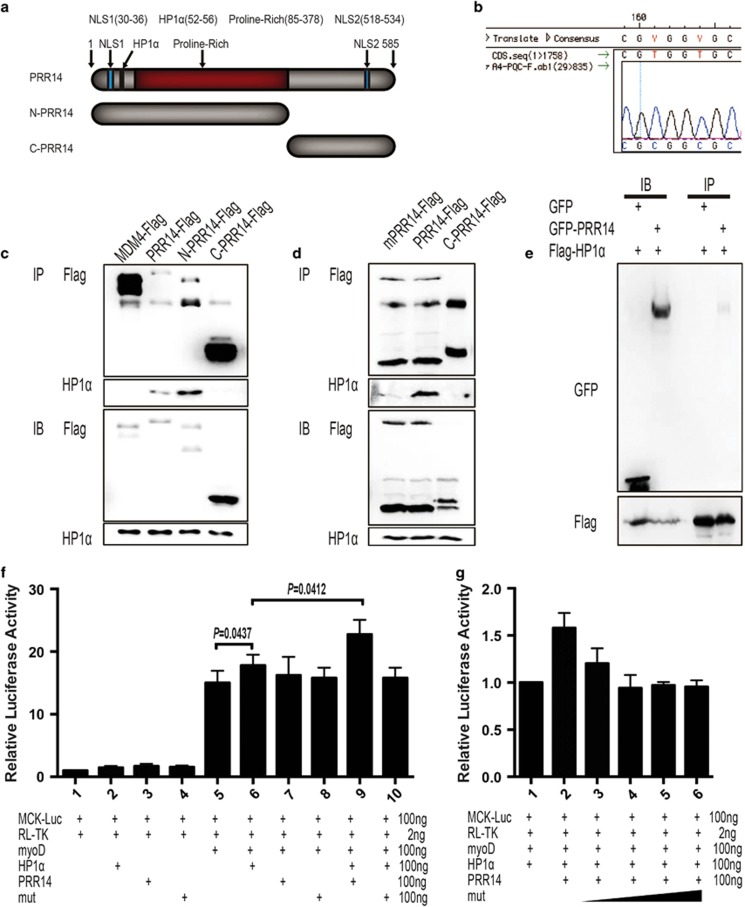
PRR14 binds to and regulates HP1*α*-mediated regulation of MyoD transcriptional activity. (**a**) Schematic diagram of the domain structure of PRR14 and the protein fragments used in this study. (**b**) Mutations were introduced into the HP1*α*-binding domain and the plasmid was sequenced to confirm the mutations. HEK293T cells were transfected with indicated plasmids. Immunoprecipitation was performed with argarose-conjugated flag-antibody (**c**–**e**). C2C12 cells cultured in GM were transfected with MCK promoter-driven luciferase reporter plasmid and pRL-TK in the presence of a combination of different plasmids as indicated. After 12 h, medium was replaced by DM to induce differentiation for 24 h. The MCK promoter activity normalized with pRL-TK was measured by dual-luciferase assay (**f**, **g**). The data are presented as mean±S.D. (*n*=5) and statistically analyzed by two-tailed student's *t-*test

HP1*α* was suggested to mediate the association between PRR14 and heterochromatin.^5^ It is, however, unclear regarding the nature of this interaction. We tested whether PRR14 might directly bind to HP1*α* by performing immunoprecipitation. Lysates from cells expressing Flag-tagged full-length, N-terminal or C-terminal constructs of PRR14 (Figure 7a) were subjected to Flag-IP. Flag-MDM4, an unrelated protein, was included as a control. The result revealed a clear binding of full-length and N-terminal PRR14, but not C-terminal PRR14 and MDM4, to HP1*α* (Figure 7c). Reciprocally, Flag-tagged HP1*α* was able to pull down co-expressed GFP-PRR14, but not GFP alone (Figure 7e). To further determine whether the putative HP1-binding domain within the N-terminus is responsible for the binding, we introduced mutations within this region (Figure 7b), which is conserved between human and mouse (Supplementary Figure S1A). The result from co-IP experiment indicated a diminished binding of the mutant to HP1*α* (Figure 7d). Together, the data indicate that PRR14 binds to HP1*α* through its N-terminal HP1-binding motif.

HP1*α* was reported to interact with MyoD and to regulate its transcriptional activity.^26^ Given that PRR14 binds to HP1*α* and MyoD induction during myoblast differentiation was greater in PRR14-expressing than control cells (Figure 4b), we asked whether PRR14 might affect MyoD transcriptional activity through its interaction with HP1*α*. We addressed this question by employing luciferase assays (E1960, Promega, Madison, WI, USA) using a luciferase reporter driven by the muscle creatine kinase (MCK) promoter, a direct target of MyoD during myogenesis.^31^ Although having little activity by itself, HP1*α* augmented the activity of MyoD, consistent with a positive regulation of MyoD transcriptional activity by HP1*α*. This result is consistent with the data shown in Figure 4b that PRR14 stimulated MyoD transcriptional activity. Interestingly, PRR14 showed little activity by itself but enhanced the effect of HP1*α* on MyoD activity. In line with its HP1*α*-binding mediated activity, the PRR14 mutant defective in HP1*α* binding failed to induce a similar effect (Figure 7f). In addition, the mutant PRR14 seemed to be able to suppress wild-type PRR14's ability to enhance MyoD activity (Figure 7g), which suggests a dominant-negative effect of this RR14 mutant.

**Figure 8:**
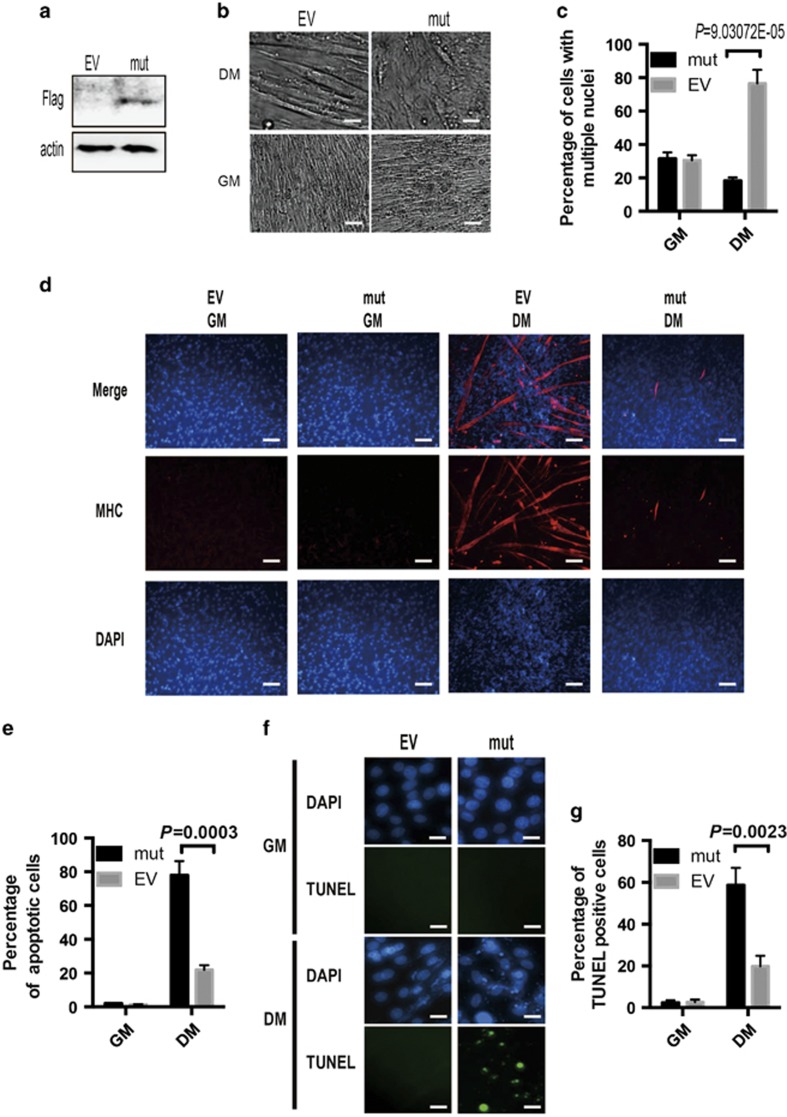
The interaction between PRR14 and HP1*α* is critical for C2C12 myogenesis. (**a**) Mutant PRR14-overexpressing C2C12 stable cell line was established and its expression was confirmed by immunoblotting. The cells were induced for myogenesis in DM for 5 days and representative phase-contrast images are shown (**b**), the percentage of cells with multiple nuclei (>4 N) was quantified by FACS and statistically analyzed (**c**). The expression of MHC was detected by immunostaining (**d**). And the statistical analysis of apoptotic cells from FACS data is presented in **e**. All FACS data are presented as mean±S.D. (*n*=3) and statistically analyzed by two-tailed student's *t-*test. (**f**) Again, cells were subjected to TUNEL assay and counterstained with DAPI. To quantify the percentage of TUNEL-positive cells, cells were counted in three different fields and 200 cells were counted for each field. The data are presented as mean±S.D. (*n*=3) and statistically analyzed by two-tailed student's *t*-test (**g**).

Considering that MyoD is a master transcription factor of myogenesis, we examined whether PRR14/HP1*α*-dependent regulation of MyoD could impact on differentiation. For this, we created C2C12 cells stably expressing the HP1*α*-binding mutant of PRR14 and assessed its differentiation potential (Figure 8a). Remarkably, expression of this PRR14 mutant almost completely prevented C2C12 myoblasts from differentiation, as demonstrated by the lack of appearance of multinucleated myotubes (Figures 8b and c), significantly diminished expression of MHC (Figure 8d), and a marked increase of apoptosis (Figures 8e–g). The results together implicate that binding of PRR14 to HP1*α* is critical for its role in MyoD-mediated C2C12 myoblast differentiation.

## Discussion

Many different mutations in *LMNA*, as well as other nuclear lamina-coding genes have been linked to several different inherited diseases with muscular dystrophy.^4, 12, 19, 20, 21^ It remains incompletely clear how mutations in these proteins, expressed in nearly all differentiated somatic cells, cause such a tissue-specific phenotype. There are currently two prevailing but nonexclusive models.^32, 33, 34, 35, 36, 37, 38, 39^ The 'mechanical stress' model is based on the observation that cells with decreased or mutated nuclear lamina protein often exhibit severe abnormalities in nuclear morphology.^32, 33, 40^ Therefore, it is hypothesized that the nuclear lamina defines the shape and mechanical properties of the nucleus. Its structural alterations may lead to increased fragility and decreased mechanical stiffness, resulting in whole-cell mechanical vulnerability.^34, 35^ During myogenesis, single-nuclear myoblasts align in parallel and fuse into multinucleated myotubes to complete the differentiation process. Such dramatic morphological alterations are associated with considerable mechanical stress. In addition to the contractile properties of muscle fibers, this is likely one of reasons why the muscle tissue is more susceptible to dysregulation of nuclear lamina-coding genes.

Consistent with its unique distribution in the lamina, siRNA-mediated *Prr14* depletion was associated with dysregulation of lamin A/C (Figure 6). Although lacking direct evidence for an interaction between PRR14 and lamin A/C, these two proteins seem to be functionally related. Poleshko *et al.*^5^ reported that siRNA-mediated lamin A/C depletion was associated with releasing PRR14 from nuclear lamina in Hela cells. Our data is also consistent with an interaction between PRR14 and lamin A/C as evidenced by the observation that Prr14 depletion resulted in marked reduction of lamin A/C abundance in C2C12 cells. Although further studies are needed to elucidate the underlying mechanism, our data suggest that PRR14 enhances myogenesis via, at least, in part maintaining the protein stability of lamin A/C and thus structure of lamina. Considering the critical role for lamin A/C in supporting the nuclear lamina structure, reduced lamin A/C abundance would likely compromise nuclear organization. The mechanical stress model seems to be in line with PRR14-mediated regulation of myogenesis.

Nuclear lamina dynamically interacts with chromatin and contributes to chromatin hierarchical organization,^36, 37^ which undergoes drastic changes during muscle-cell differentiation.^38^ The second model or 'gene expression' model proposes that, in addition to its structural and mechanical function, nuclear organization has a role in differentiation through regulation of tissue-specific gene expression. We showed that PRR14 augmented the transcriptional activity of MyoD via directly binding to HP1*α*, a constitutive component of heterochromatin involved in regulation of gene expression and higher-order chromatin structure.^39^ It was previously reported that siRNA-mediated depletion of *Hp1α* impairs myogenesis in C2C12 cells,^25, 26^ and HP1*α* participates in not only reorganization of heterochromatin but selective regulation of tissue-specific genes during neuron cell differentiation.^41^ The PRR14/HP1*α* interaction-induced MyoD stimulation uncovered by the present study offers novel insight into the gene expression model in regulation of myogenesis.

The *PRR14* gene is located in 16p11.2. There were only eight reported cases with copy number variations (deletions) surrounding this locus. Six out of the eight cases have shown signs of developmental delay. Interestingly, hypotonia underlies delay in two out of four children with delayed mobility skills.^42^ Further study is warranted to investigate the association of *PRR14* with human laminopathies.

## Materials and Methods

### Cell culture, transfection and stable cell lines establishment

C2C12 and HEK293T were maintained using standard conditions. C2C12 cells were induced to differentiate into myotubes in differentiation medium (DMEM high glucose supplemented with 4 mM glutamine, 100 IU ml^−1^ penicillin, 100 *μ*g ml^−1^ streptomycin, and 2% (v/v) horse serum). DNA transfection was carried out using Lipofectamine 2000 (Invitrogen, Carlsbad, CA, USA). siRNA transfection was mediated by Lipofectamine RNAiMAX Transfection Reagent (Invitrogen).

Retroviral production and infection were done following the protocol from Clontech. Cells were allowed to recover for 24 h prior to selection with hygromycin for 1 week. Cells transduced with the empty vector were also established and used as control cell lines.

### Plasmids

cDNA encoding full-length human PRR14 was purchased from DF/HCC DNA Resource Core (Boston, MA, USA) and cloned into pQCXIH (Clontech) for retrovirus production and pEGFP-N1 (Clontech) by Gateway Cloning system (Invitrogen). For deletion mutation, the full-length plasmid was used as template for PCR with the following primers: NPRR14-F: CGGAATTCCCATGGACTTGCCCGGGGAC, NPRR14-R: CTAGTCTAGACGAGGGGGTGGCGGGGCTCG, CPRR14-F: CGGAATTCCCATGCCCTGTCTCCGGAAAGAGG, CPRR14-R: CTAGTCTAGAGGTCCAGTGGGGCTG. Site-directed mutation was introduced by Q5 Site-Directed Mutagenesis Kit (NEB, Ipswich, MA, USA) using primers: PRR14a-F: GTCCTGGCCGCGGCGCTAGAAGATG and PRR14a-R: CCGCCGAGAGGCCTTTTC.

All plasmids were sequenced to confirm the insertion. For dual-luciferase assay, plasmids MCK-luciferase (#16062, addgene, Cambridge, MA, USA), pRL-TK (Promega), myc-MyoD (#8399, addgene) and GFP-HP1*α* (#17652, addgene) were purchased.

### RNA preparation, reverse transcription and qPCR

Total RNA was extracted using the standard TRIzol method (Life Technologies, Grand Island, NY, USA) and used for the first-strand cDNA synthesis by iScript cDNA Synthesis Kit (Bio-Rad, Hercules, CA, USA). Real-time PCR was performed with SYBR Green JumpStart Taq ReadyMix (Sigma) using the StepOnePlus Real-Time PCR System (Bio-Rad). All reactions were run in duplicate. After vortexing, 10 *μ*l aliquots of the mixture were pipetted into each well of a 96-well thin-wall PCR plate (Bio-Rad). PCRs consisted of a denaturing cycle at 94 °C for 2 min, followed by 40 cycles of 15 s at 94 °C and 1 min at 60 °C. Relative mRNA amounts of target genes were calculated after normalization to an endogenous reference gene (18 s) and relative to the negative control with the arithmetic formula 2^−ΔΔCt^. The following primer sequences were used for qPCR: mPRR14-F: TTCACACCTAACAAAACACCACA, mPRR14-R: CCTCCGAGATGGCAATCTTCA, 18 s-F: GTAACCCGTTGAACCCCATT, 18 s-R: CCATCCAATCGGTAGTAGCG, Lmna-F: GGATGCTGAGAACAGGCTACA, Lmna-R: CTCTCGCTGCTTCCCGTTATC, HP1*α*-F: GATCATCGGAGCAACAGATTCC, HP1*α*-R: CGTGCCACGTCAGTCTCTC.

### Cell cycle analysis

C2C12 cells were trypsinized, collected by centrifugation (500 g, 5 min), washed in 4 °C PBS once and gently resuspended in 1 ml hypotonic fluorochrome solution (PI 50 *μ*g ml^−1^ in 0.1% sodium citrate, 10 mM NaCl, 0.3% NP-40 plus 25 mg ml^−1^ RNase) in polypropylene tubes, followed by incubation in 37 °C for 30 min. The tubes were placed at 4 °C in darkness for flow cytometric analysis.

### Immunohistochemistry, western blotting and immunoprecipitation

For western blot, cells were lysed in NP-40 buffer (150 mM NaCl, and #8232; 1% NP-40, 50 mM Tris-HCl, pH 8.0, 0.1% SDS) supplemented with phosphatase and protease inhibitors (1 mM Na3VO4, 1 mM NaF, 1 mM PMSF, 1 mM EDTA, 1 *μ*g ml^−1^ aprotinin, 1 *μ*g ml^−1^ leupeptin). Western analysis was performed using standard procedures and the protein of interest was normalized to *β*-actin protein level. For immunohistochemistry, cells were fixed in ice cold 1 : 1 Acetone/methanol fixative, and blocked with 1% BSA/1% Triton X-100/1 X PBS. The following antibodies were used: Flag (clone M5, Sigma), HP1*α* (#2616, Cell Signaling, Beverly, MA, USA), MHC (clone A4.1025, Millipore, Temecula, CA, USA), lamin A/C (#4777; Cell Signaling, Danvers, MA, USA), MyoD diluted at 1 : 100 (sc-760; Santa Cruz, Santa Cruz, CA, USA), PRR14 (ab174532, abcam, Cambridge, MA, USA), Histone H3 (tri methyl K9; ab8898, abcam), lamin B1 (sc-6216; Santa Cruz) and *β*-actin (clone ab8226; abcam). Immunoprecipitation was done in HEK293T cells. After 48 h transfection, cells were collected and lysed in RIPA buffer (25 mM Tris-HCl, (pH 7.6), 150 mM NaCl, 1% NP-40, 1% sodium deoxycholate, 0.1% SDS) and incubated with flag beads (Anti-FLAG M2 magnetic Beads, Sigma). Immunoprecipitation was performed following the protocol.

### Statistical analysis

The data are presented as mean±S.D. Two-tailed student's *t-*test was used to determine the significance of the difference between the two groups. If not indicated otherwise, the criterion for significance was set at *P*⩽0.05 (Prism 6, GraphPad Software, San Diego, CA, USA).

## Abbreviations

DM: differentiation medium
PRR14: proline rich 14
MHC: myosin heavy chain
MyoG: myogenin
MyoD: myogenic differentiation 1
HP1*α*: heterochromatin protein 1 alpha
Lmna: lamin A
MCK: muscle creatine kinase
EV: empty vector
Mut: mutant PRR14
