# Supplementary material for: A novel role of PRR14 in the regulation of skeletal myogenesis
Source: Cell Death Dis. 2015 Apr 23;6(4):e1734–. doi: 10.1038/cddis.2015.103 (PMC4650536; doi:10.1038/cddis.2015.103)
Supplement: Supplementary Figure 1 [file cddis2015103x1.docx]

Supplementary Figure S1 Yang 2015


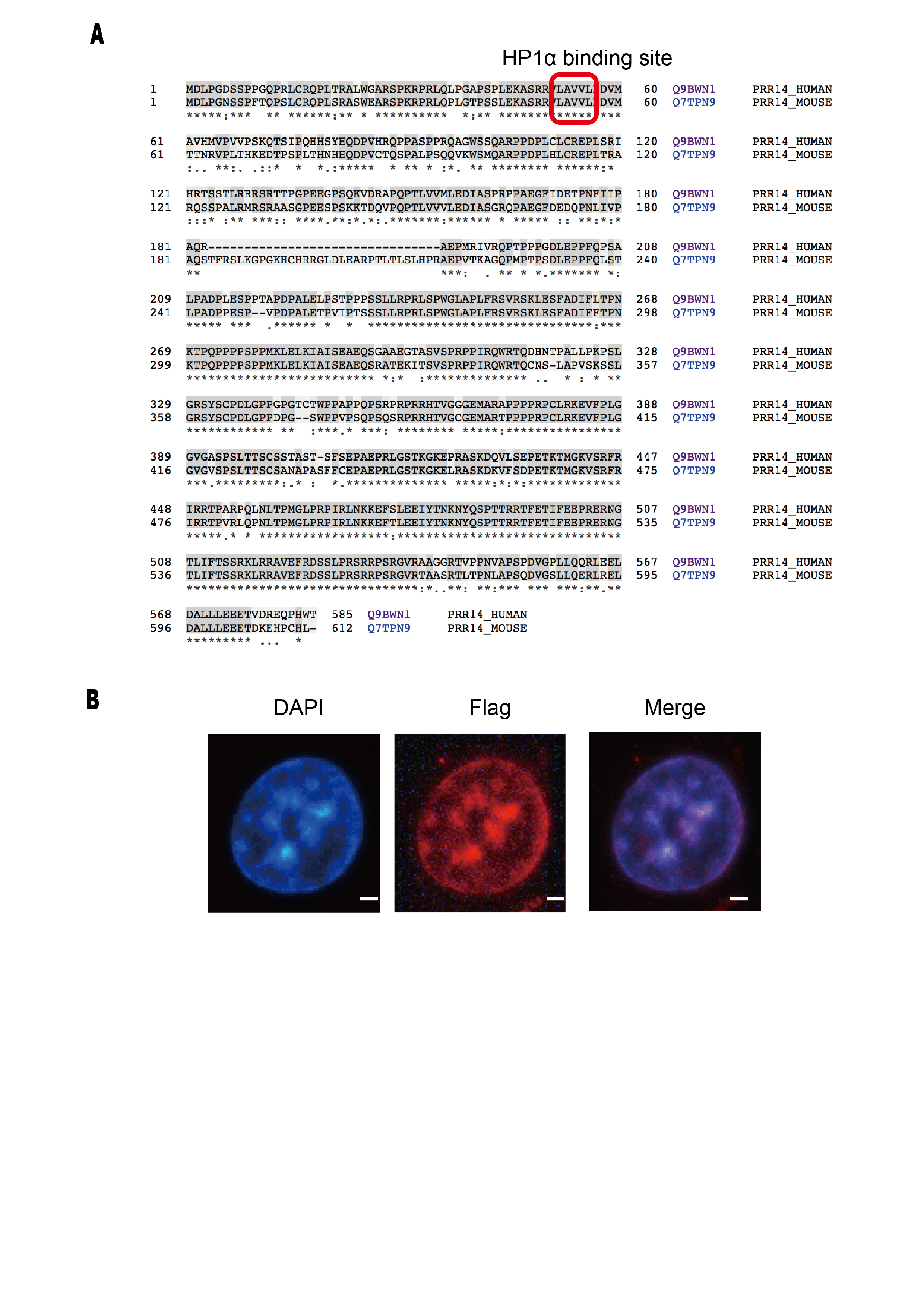


**Supplementary Figure Legends**

PRR14 is highly conserved between human and mouse.

(A) Alignment of protein sequences from human and mouse. Reference sequences were retrieved from NCBI database and pairwise alignment was performed via UniProt. Predicted HP1a binding site is boxed. (B) Human PRR14 protein subcellular location in C2C12 cells was detected by Immunofluorescence analysis with flag antibody. 24h after transient transfection, C2C12 cells were harvested for immunostaining with flag antibody and DAPI.
